# Etiological Profiles of Patients With Thrombocytopenia in Central India: A Tertiary Centre Study

**DOI:** 10.7759/cureus.32571

**Published:** 2022-12-15

**Authors:** Deepshikha Verma, Ranjan Yadav, Varsha Rampuri, Rajni Choudhary, Abhiram Awasthi

**Affiliations:** 1 Department of Pathology, Gandhi Medical College and Hamidia Hospital, Bhopal, IND; 2 Department of Orthopaedic Surgery, Datta Meghe Institute of Medical Sciences, Wardha, IND

**Keywords:** leukaemia, bone marrow morphology, megakaryocytes, bone marrow aspiration, thrombocytopenia

## Abstract

Introduction

Platelet-related disease may result from an abnormal platelet count, namely thrombocytopenia or thrombocythemia, or altered platelet function, and thus is associated with bleeding or with thrombotic manifestation. Thrombocytopenia is defined as a subnormal number of platelets i.e. less than 1,50,000/µL in the peripheral blood. It can lead to inadequate clot formation and increased risk of bleeding and is a common indication for bone marrow aspiration and biopsy.

Methodology

The study was a hospital-based prospective observational study from January 2019 to June 2020. All cases of thrombocytopenia which were diagnosed first on haematology analyser (platelet counts <150,000/µL) and confirmed subsequently by peripheral smear with/without bleeding manifestations due to thrombocytopenia were taken up for the study. The aims and objectives of this study were to find out the epidemiological spectrum and prevalence of thrombocytopenia according to age and sex along with the correlation of haematological and bone marrow findings of such patients.

Result

This study comprised 100 cases of thrombocytopenia, with acute leukaemia accounting for the majority (28/100), followed by dimorphic anaemia (15/100), megaloblastic anaemia (11/100), hypocellular marrow, infection, and other conditions. All cases (100%) displayed the clinical symptom of widespread weakness and pallor, which was followed by fatiguability (72%) and dyspnoea (48%). Many thrombocytopenic individuals also had lymphadenopathy and hepatomegaly, the last two least common appearances. Lymphadenopathy and hepatomegaly which were the last two least common presentations were present in a significant number of thrombocytopenic patients.

Conclusion

The study of bone marrow is helpful in the diagnosis of thrombocytopenia cases. Bone marrow examination is a simple, safe outpatient procedure and yields an impressive amount of diagnostically valuable data in a wide variety of disorders of thrombocytopenia. An evaluation of the patient's bone marrow unquestionably aids in the early diagnosis and treatment of their ailment.

## Introduction

A decrease in the peripheral blood platelet counts below the lower normal limit of 150,000/µL is referred to as thrombocytopenia [1]. It may result in poor clot formation and is associated with a higher risk of bleeding [2]. It is frequently used as a reason for bone marrow biopsy and bone marrow aspiration. Hypo-proliferation in the bone marrow or platelet breakdown in the periphery can both cause thrombocytopenia. The testing of the bone marrow is frequently used to distinguish between these two groups. The range of the normal platelet value is 150,000 to 450,000/µL. Thrombocytopenia is defined by platelet counts less than 150,000/µL; however, these numbers do not show the underlying pathophysiology [3]. Thrombocytopenia can be classified into three stages which include a mild category having platelet count between 100,000 - 150,000/µL, moderate thrombocytopenia having platelet count between 50,000 - 100,000/µL and severe thrombocytopenia having platelet count < 50,000/µL [3].

Thrombocytopenia is a significant finding in any hospital setting that is seen in numerous conditions with variable spectrums. Its severity ranges from an incidental finding otherwise undetected to severe life-threatening bleeding. So the early detection of the cause of thrombocytopenia followed by proper treatment may prove lifesaving. A bone marrow examination is a helpful and affordable diagnostic method in haematological practice. Both neoplastic and non-neoplastic haematological conditions are identified using the aid of a bone marrow aspiration. This additionally, is used for the classification of anaemia, and the assessment of several cytopenias and unidentified pyrexia. Typically, a bone marrow evaluation requires two distinct but connected samples. The first is a cytological preparation of bone marrow cells by aspirating the marrow and smearing the cells, providing excellent cell morphology visualisation and cellular element enumeration. The second specimen is a bone and associated marrow needle biopsy that accurately assesses the bone marrow's cellularity, fibrosis, infections, or infiltrative illnesses as well as cellular distribution and ratios. The present study has been undertaken to study the cause of thrombocytopenia based on bone marrow findings and haematological parameters. Morphological examination of megakaryocytes in bone marrow aspiration in diagnosing various haematological disorders leading to thrombocytopenia was also evaluated in the current study.

## Materials and methods

This study was a hospital-based prospective observational study from January 2019 to June 2020. It was approved by Institutional Ethics Committee, Gandhi Medical College Bhopal with IEC reference number: 637/MC/IEC/2020. All the cases which were diagnosed first on a hematology analyzer as thrombocytopenia (platelet counts <150,000/µL) and also confirmed subsequently by peripheral smear with/without bleeding manifestations due to thrombocytopenia were taken up for the study while patients with bleeding manifestations other than thrombocytopenia, those patients with thrombocytopenia where bone marrow aspiration is not indicated and non-co-operative patients were excluded from this study. For each case, signed consent was obtained. All necessary clinical information was gathered. Then, a bone marrow aspiration was carried out for every case using all aseptic techniques. The bone marrow aspiration smears were then stained with Leishman stain and as per standard guidelines these smears were examined and findings were documented.

A fresh 4ml sample was withdrawn in an ethylene diamine tetra acetic acid (EDTA) vial and a peripheral smear was prepared with fresh blood. Leishman’s stain was poured on the slide and waited for 2 minutes. This allowed the fixation of peripheral blood film in methyl alcohol, a double quantity of buffered water was added dropwise over the slide and mixed for 5 minutes. After washing it in water for 1-2 minutes, the slide was air-dried and then examined in an oil immersion lens [4-6].

Bone marrow aspiration was done from the sternum or posterior superior iliac spine (PSIS), taking care of all aseptic precautions. Local anesthesia (2-5ml of lignocaine) was given to minimize discomfort. After 2-3 minutes the bone marrow aspiration needle was passed perpendicularly at the site. The stellate was removed, the needle was attached, and about 1ml or 2ml of bone marrow was aspirated. Smears were drawn and air dried from the aspirated material and stained with Leishman’s stain same as for peripheral blood film and then smears were examined in a high power field [4-6].

Several megakaryocytes were expressed as the number per 10 low power fields (LPFs) and results were further subdivided into absent (0/10 LPFs), decreased (1/5-10LPFs), normal (1/1-3 LPFs), and increased (>2/LPF) [7].

## Results

During the study period, a total of 100 cases with thrombocytopenia underwent bone marrow examination in the hematology section of the Department of Pathology. All the patients had their bone marrow aspiration.

Hematological diseases can affect both children and adults on a very broad spectrum. To make the final diagnosis, a bone marrow aspiration/biopsy is a valuable test. It is one of the most common and very safe invasive procedures which is performed regularly in hospitals. Even in cases of severe thrombocytopenia, an invasive surgery can be easily carried out. Commonly it is done for the evaluation of unexplained cytopenia and leukemia and diagnosing storage disorders, and also at times for staging of leukemias/other malignancies.

This study comprised 100 cases of thrombocytopenia, with acute leukemia accounting for the majority (28/100), followed by dimorphic anemia (15/100), megaloblastic anemia (11/100), hypocellular marrow, infection, and other conditions (Table 1). All cases (100%) displayed the clinical symptom of widespread weakness and pallor, which was followed by fatiguability (72%) and dyspnoea (48%). Many thrombocytopenic individuals also had lymphadenopathy and hepatomegaly, the last two least common appearances. Lymphadenopathy and hepatomegaly which were the last two least common presentations were present in a significant number of thrombocytopenic patients (Table 1).

**Table 1:** Incidence of diseases in the present study

Name of disease	Number of patients
Acute leukemia	28
Dimorphic anemia	15
Megaloblastic anemia	11
Immune thrombocytopenic purpura	9
Reactive to infection	9
Hypocellular marrow	9
Granulomatous disease	4
Chronic myeloid leukemia	3
Myelofibrosis	2
Myeloproliferative disorders (MPD)	2

Of the 100 cases, thrombocytopenia was commonly seen in males (57%) as compared to females (43%). A wide range of age distribution was seen in the present study with a peak in 11-20 years of age. Hypercellular marrow was most common 50/100 (50%), normocellular marrow was seen in 18/103 (18%), and hypocellular marrow in 31/100 cases (31%) while one case had a dry tap. Splenomegaly was present in 47% of cases while hepatomegaly was seen in 27% of cases.

One hundred percent of cases of immune thrombocytopenic purpura (ITP) had an increased number of megakaryocytes followed by 33% cases of dimorphic anemia. Dysplastic changes in megakaryocytes were found in 35% of cases. The most common dysplastic feature observed was multiple separate nuclei (26%) commonly observed in ITP (77.77%). Multiple separated nuclei were most commonly seen in ITP followed by dimorphic anemia while micro-megakaryocytes were most commonly seen in ITP followed by dimorphic anemia and hypogranular form was most commonly observed in infection.

Non-dysplastic changes in megakaryocytes were observed in 65% of cases. The immature form was most commonly seen in ITP whereas emperipolesis was seen in all cases of ITP. Again, cytoplasmic vacuolation was most commonly seen in ITP followed by dimorphic anemia while smooth borders, bare nuclei, and hypolobated form were most commonly seen in ITP (Table 2).

**Table 2:** morphological alteration in megakaryocytes in various hematological disorders. MPD- myeloproliferative disorder

Name of disease	Number of cases	Multiple separated nuclei	Micro/Megakaryocytes	Hypogranular form	Immature form	emperiopolesis	Cytoplasmic vacuolation	Smooth borders	Bare nuclei	Dysmorphic Form	Hypolobated form
Acute leukemia	28	2	-	-	-	-	-	-	-	-	-
Dimorphic anemia	15	8	4	1	2	3	6	4	3	1	7
Megaloblastic anemia	11	3	3	1	-	3	1	2	3	1	6
Immune thrombocytopenic purpura	9	6	4	2	5	9	7	4	4	2	6
Reactive to infection	9	2	2	3	-	2	1	2	-	-	1
Hypocellular marrow	9	-	1	-	-	1	-	1	1	-	-
Granulomatous disease	4	1	-	-	-	1	-	-	-	-	-
Chronic myeloid leukemia	3	-	1	-	-	1	-	-	1	-	-
Myelofibrosis	2	-	2	-	2	-	-	2	-	2	2
MPD	2	1	1	1	-	-	-	-	-	-	-

## Discussion

The most common age group in this study was 11-20 years old (29%), with a male preponderance of 57%. Several other studies found that the most common age groups were 21-30 years old, 15 years old, and 51-60 years old with a male preponderance in all studies [8-10]. There were 100 cases of thrombocytopenia in this research predominantly due to acute leukemia (28%) followed by dimorphic anemia (15%) and megaloblastic anemia (11%). Gupta et al. [11] in their study found that ITP was the most common cause of thrombocytopenia, followed by megaloblastic anemia and iron deficiency anemia, while Choudhary et al. [12] concluded that the most common cause was megaloblastic anemia followed by acute leukemia and ITP. According to Muhury et al. [13], the most common cause of thrombocytopenia was acute myeloid leukemia (AML), followed by ITP and acute lymphoid leukemia (ALL). In the study by Pokheral et al. [14], megakaryocytic thrombocytopenia was the most common cause while in Ratnakar et al. [15] study it was megaloblastic anemia. Sandeep et al. [16] concluded megaloblastic anemia was the most common cause of thrombocytopenia in their study while it was third most common cause in the present study.

Out of 28 cases of acute leukemia in the present study, 12 were acute leukemia not specified morphologically while 10 were ALL, four were AML and two were acute promyelocytic leukemia (APML). Male preponderance was seen in cases of acute leukemia with 18 males out of 28 cases. Similar male preponderance was seen in Yadav et al. [17] and Girish et al. [18]. According to the research by Lim and Ifthikharuddin [19], bone marrow suppression by chemotherapeutic agents and platelet sequestration in the spleen also contribute to thrombocytopenia in lymphoma along with immune-mediated platelet destruction and decreased platelet production when the marrow is involved in lymphoma.

A total of 15 cases were reported as dimorphic anemia in the present study with a peak at 11-20 years and 21-30 years showing male predominance (53%). The age ranges 11-20 years and 21-30 years had the highest frequency at 33.34% in each age group. Dimorphic anemia was identified in 18.58% and 67.74% of patients in two similar studies with a male preponderance in both [10,17].

A total of 11 cases were reported as megaloblastic anemia in the present study with a male-to-female ratio of 4.5:1. The most impacted age group was 41-50 years (36.36%), followed by 31-40 years (27.27%). A broad age range with a male majority was seen in other studies too [20,21]. The majority of patients had generalized weakness associated with fever. Pallor was present in all of the patients. For a definite diagnosis, a bone marrow aspiration was performed. It agreed with research conducted by Muhury et al. [13]. It is generally believed that as the severity of anemia increases, thrombocytopenia develops followed by neutropenia [22]. The cause of thrombocytopenia in megaloblastic anemia has been proposed as hypoproduction whereas bone marrow shows decreased megakaryocytes and platelet indices are studied including cases of megaloblastic anemia under the category of hypoproduction [23].

Out of nine cases of ITP, female preponderance was seen (66.7%) and the most common age group involved was 21-30 years. All nine (100%) had an increased number of megakaryocytes in bone marrow smears which was similar to the observations of Choudhary et al. [12] and Muhury et al. [13]. In our study, emperipolesis was a striking feature in ITP shown by 100% cases which was similar to Muhury et al. [13] and Choudhary et al. [12]. Tirumalasetti et al. [24], in their study, concluded that the common dysplastic feature in megakaryocytes of ITP observed was micromegakaryocytes (57.1% cases) similar to observations made by Shi et al. [25] while in contrast, Gupta et al. [11] observed that the most common morphological alteration found in cases of ITP were hypolobulation and hypogranular forms.

As a tertiary care facility located in the state capital, it serves the typical population surrounding it, which is typically urban, sub-urban, and close to the peripheral area. However, a sizable portion of the periphery is unscreened for this study. The results may differ if the study had a larger sample size because this one had a relatively small sample size.

## Conclusions

The leading causes of thrombocytopenia found in our study were acute leukemia which on early diagnosis can get better treatment followed by dimorphic anemia which can be treated with minimum expenditure when diagnosed correctly. Bone marrow examination is a very important tool in the diagnosis and helps in the management of hematological disorders. Bone marrow aspiration turns out to be a very simple and safe investigation that could be repeated if needed and carried out in outpatients as well. 

The study of bone marrow forms the mainstay in the diagnosis of thrombocytopenia cases. Bone marrow examination is a simple, safe outpatient procedure and yields an impressive amount of diagnostically valuable data in a wide variety of disorders of thrombocytopenia. We can continue to learn more about the pathogenesis of various hematopoietic disorders by conducting additional research on megakaryocytic alteration and its role in thrombocytopenia. These studies may also point to broader clinical uses for the more recent techniques for controlling platelet count and function. An evaluation of the patient's bone marrow unquestionably aids in the early diagnosis and treatment of their ailment.
